# Investigation of Micro- and Nanosized Particle Erosion in a 90° Pipe Bend Using a Two-Phase Discrete Phase Model

**DOI:** 10.1155/2014/740578

**Published:** 2014-10-14

**Authors:** M. R. Safaei, O. Mahian, F. Garoosi, K. Hooman, A. Karimipour, S. N. Kazi, S. Gharehkhani

**Affiliations:** ^1^Department of Mechanical Engineering, Faculty of Engineering, University of Malaya, 50603 Kuala Lumpur, Malaysia; ^2^Department of Mechanical Engineering, Faculty of Engineering, Ferdowsi University of Mashhad, Mashhad, Iran; ^3^Department of Mechanical Engineering, University of Semnan, Semnan, Iran; ^4^School of Mechanical and Mining Engineering, The University of Queensland, St Lucia, Brisbane, QLD 4072, Australia; ^5^Department of Mechanical Engineering, Najafabad Branch, Islamic Azad University, Isfahan, Iran

## Abstract

This paper addresses erosion prediction in 3-D, 90° elbow for two-phase (solid and liquid) turbulent flow with low volume fraction of copper. For a range of particle sizes from 10 nm to 100 microns and particle volume fractions from 0.00 to 0.04, the simulations were performed for the velocity range of 5–20 m/s. The 3-D governing differential equations were discretized using finite volume method. The influences of size and concentration of micro- and nanoparticles, shear forces, and turbulence on erosion behavior of fluid flow were studied. The model predictions are compared with the earlier studies and a good agreement is found. The results indicate that the erosion rate is directly dependent on particles' size and volume fraction as well as flow velocity. It has been observed that the maximum pressure has direct relationship with the particle volume fraction and velocity but has a reverse relationship with the particle diameter. It also has been noted that there is a threshold velocity as well as a threshold particle size, beyond which significant erosion effects kick in. The average friction factor is independent of the particle size and volume fraction at a given fluid velocity but increases with the increase of inlet velocities.

## 1. Introduction

Erosion-corrosion, defined as the accelerated corrosion following the damage of surface films, is a common cause of failure in a large amount of power plant equipment like pipes, pumps, compressors, vessels, and turbines. It can often be assumed that corrosion is controlled by adjusting the mass transfer while erosion is under the flow of a particulate second phase. This is a credible assumption as corrosion films are brittle-like materials and therefore are eroded easily by impacting particles [1, 2]. This phenomenon has been investigated experimentally in a number of pioneering studies; see [3–7], for instance. Despite recent advances in computational techniques, erosion-corrosion process is yet to be fully resolved with reasonable accuracy. A multitude of reasons for this rather slow development of simulation techniques applied to this problem can be mentioned. For modeling mass transfer near the solid boundaries, it is necessary to solve the governing equations across the mass transfer boundary layer. In aqueous flows this layer may be an order of magnitude shorter than the viscous sublayer. This requires fine meshes in the near-wall region. Utilizing fine near-wall grids with the support of appropriate near-wall turbulence models, the required mass transfer data for corrosive species can be evaluated [8].

Chen et al. [9] studied erosion prediction approach and its usage in oilfield fittings, especially 3-D elbows and plugged tees, using CFX which is a commercially available CFD package. They used RNG *k*-*ε* turbulence model along with DPM to track the particles. The results demonstrated that particle rebound and erosion profile have the most significant roles in particles motion inside oilfield geometries. The comparisons also indicated that CFD predictions for erosion are in good agreement with experimental data.

An erosion prediction approach for specifying wear profiles for a 2-D jet impingement test has been developed by Gnanavelu et al. [10]. This prediction model was according to material wear data achieved from laboratory experiments and CFD modeling. They found an appropriate relationship between predicted and experimental data. Although they found that due to some assumptions about particle size and shape, material hardening, numerical errors, and so forth, some essential errors always exist in the calculation.

Mohyaldin et al. [11] have used three methods (empirical, semiempirical, and computational fluid dynamics, i.e., CFD) to model 2-D sand erosion in a pipe, a problem with significant practical application in oil and gas industry. The results of this study have shown that the direct impingement model (semiempirical model) agrees with the results achieved from the discrete phase model (DPM) implemented in CFD whereas the CFD results dramatically underpredict the empirical ones.

Particles, in an erosion problem, can be external to fluid flow; that is, they may be removals from the walls or upstream flow processes. There are, on the other hand, cases where particles are internal to flow like nanofluids. Nanofluids are synthesized by adding highly conductive solid materials to the base fluid, such as water, ethylene glycol, and oil, all with relatively lower thermal conductivity, usually to improve the heat transfer performance of the mixture (compared to that of the base fluid) [12–14]. The idea of adding microparticles to base fluids was presented decades ago; however, microsized particles have the tendency to settle in the suspension, thereby potentially leading to adverse effects. Use of nanofluids, with nanosized particles suspended in the base fluids, would mitigate the issues of fouling and pipe blockings. In addition, the presence of microsized abrasive solid materials will cause erosion and corrosion of pipes and damage pumps and other devices [15].

Routbort et al. [16] have investigated the effect of nanoparticles on erosion in a car radiator. The nanofluids in their study were 1–4% (volume) silicon carbide (SiC) in water as well as 0.1–0.8% (volume) cupric oxide (CuO) in ethylene glycol. Experiments were conducted in the range of 4 m/s–10 m/s (for velocities) and at 90°–30° impact angles. The radiator was made of Al3003 typical radiator material. In their tests, they did not observe any erosion using nanofluids. Just in one case (Cu/water nanofluid, velocity of 9.6 m/s and impact angle of 90°) the galvanic pitting (and not erosion) was observed. In this case, the material loss rate due to galvanic pitting was around 4 × 10^−2^ *μ*m/hr which indicated that the erosion had the least effect.

In a subsequent study, Routbort et al. [17] have studied the erosion of nanofluids on impeller of a cast aluminum car cooling system. They used 0.1–0.8% (volume) CuO in ethylene glycol and 0.5–4.0% (volume) SiC in water and in ethylene glycol/water (50%-50%) mixture as nanofluids. The experiments were conducted in the range of 2 m/s–10 m/s (for velocities) and at 90°–30° impact angles. The impeller was made of Al3003 material. Their study has shown no weight loss measured after testing 2% (volume), 170 nm SiC/water for more than 700 hours at 8 m/s velocity, that is, no damage to the impellor of a commercial automobile water pump.

However, in their latest report, Routbort et al. [18] have found 0.65% erosion of impeller after hundreds of hours of pumping SiC/water and SiC/ethylene glycol-water (50/50 vol.%) nanofluids at high mass flow rates (20–28 L/min).

In view of the above, comprehensive analysis of nanofluids as erosive materials is yet missing in the literature [19, 20]. In particular, erosion of nanofluids in turbulent flow regime inside industrial fittings is not fully understood. Hence, the present study aims at investigating turbulent flow of dilute water/Cu micro- and nanofluids in a 3-D 90° elbow using finite volume method with standard *k*-*ε* turbulence and DPM. The simulation results for microsized particle flow regime are compared with those in the literature for validation purpose. Special attention was paid to micro- and nanosized copper particles of different solid volume fractions and Reynolds numbers in a commercial elbow.

## 2. Governing Equations of Turbulent Micro- and Nanofluids Erosion

The underlying physical assumption in this study is that the particles are carried by the flowing fluid. Therefore, continuity, momentum, DPM, and turbulent equations are used to analyze the flow. The spherical particles' velocity is assumed to be the same as those of flowing fluid. Assuming constant thermophysical properties for fluid and particles, the governing equations are as follows [21–23].

Continuity equation:

(1) $$\begin{array}{c} \frac{\partial }{\partial t}\left(\rho \right)+\nabla \cdot \left(\rho \vec{V}\right)=0. \end{array}$$

Momentum equation:

(2) $$\begin{array}{c} \frac{\partial }{\partial t}\left(\rho \vec{V}\right)+\nabla \cdot \left(\rho \vec{V}\vec{V}\right)=-\nabla P+\nabla \cdot \left[\mu \left(\nabla \vec{V}+\nabla {\vec{V}}^{T}\right)\right]+\rho g. \end{array}$$

Standard *k*-*ε* turbulence model is as follows.

Turbulent kinetic energy transport equation:

(3) $$\begin{array}{c} \frac{\partial \left(\rho k\right)}{\partial t}+\nabla \cdot \left(\rho \vec{V}k\right)=\nabla \cdot \left[\left(\mu +\frac{\mu_{t}}{\sigma_{k}}\right)\nabla k\right]+G_{k}-\rho \varepsilon . \end{array}$$

Dissipation of turbulent kinetic energy transport equation:

(4) ∂(ρε)∂t+∇·(ρV→ε)  =∇·[(μ+μtσε)∇ε]+εk(Cε1Gk−ρεCε2).

The turbulent eddy viscosity obtained from Prandtl-Kolmogorov relation:

(5) $$\begin{array}{c} \mu_{t}=C_{\mu }\rho \frac{k^{2}}{\varepsilon }. \end{array}$$

The turbulence kinetic energy production of the mean velocity gradients, *G*_*k*_, is given as:

(6) $$\begin{array}{c} G_{k}=\mu_{t}\nabla \vec{V}\cdot \left(\nabla \vec{V}+\nabla {\vec{V}}^{T}\right)-\frac{2}{3}\nabla \cdot \vec{V}\left(3\mu_{t}\nabla \cdot \vec{V}+\rho k\right). \end{array}$$

The constants for the standard *k*-*ε* turbulence model in the above formula are represented in Table 1 [24, 25].

DPM is as follows:

(7) $$\begin{array}{c} m_{p}\frac{d{\vec{v}}_{p}}{dt}=\sum \vec{F}, \end{array}$$

where $\vec{F}$ is an external force acting on the particles which for fine particles with high density ratio (more than one) is drag and buoyancy forces [26].

Therefore, the equation of motion can be simplified to the following form:

(8) $$\begin{array}{c} \frac{d{\vec{v}}_{p}}{dt}=F_{D}\left(\vec{v}-{\vec{v}}_{p}\right)+\frac{g\left(\rho_{p}-\rho \right)}{\rho_{g}}, \end{array}$$

where [27]

(9) $$\begin{array}{c} F_{D}=\left(\frac{18\mu }{\rho_{p}d_{p}^{2}}\right)\left(\frac{C_{D}\text{R}{\text{e}}_{p}}{24}\right), \end{array}$$

wherein *Re*_*p*_ is the particle Reynolds number and is given as [28–30]

(10) $$\begin{array}{c} \text{R}{\text{e}}_{p}=\left(\frac{\rho d_{p}\left|{\vec{v}}_{p}-\vec{v}\right|}{\mu }\right). \end{array}$$

The drag coefficient, *C*_*D*_, as a function of the particle Reynolds number is defined by [31, 32]

(11) CD=24Re(1+11.2355Re0.653)+(−0.8271)Re8.8798+Re.

The solid particle erosion rates are defined as [33, 34]

(12) $$\begin{array}{c} R_{\text{erosion}}=\overset{N}{\underset{p=1}{\sum }}\left(\frac{{\dot{m}}_{p}C\left(d_{p}\right)f\left(\alpha \right){\text{v}}^{b(\nu )}}{A_{f}}\right), \end{array}$$

where *C*(*d*_*p*_) is a function of particle diameter, *f*(*α*) is a function of impact angle, *α* is the angle between the particle trajectory and wall, v is the relative velocity among particles, *b*(*ν*) is a function of relative velocity among particles, and *A*_*f*_ is the cell face area at the wall [33].

## 3. Boundary Conditions

**Figure 1:**
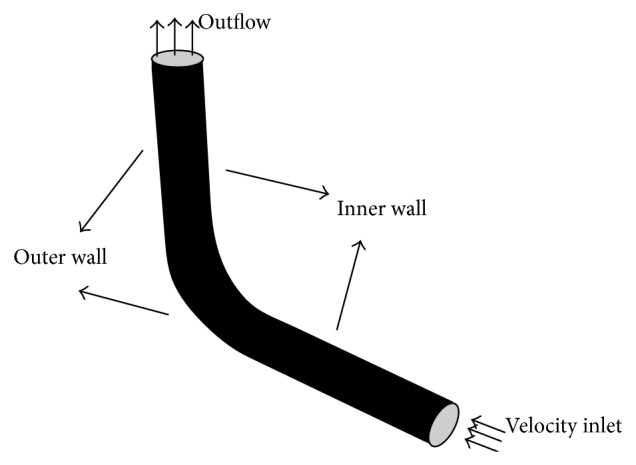
Schematic description of the pipe flow configuration with the elbow being considered for analysis.

Figure 1 illustrates the schematic of the problem which is analyzed in the present study. The boundary conditions are also indicated in this figure.

## 4. Numerical Method

The FLUENT commercial code based on finite volume method which has been used in some previous works [21, 22, 35–37] was applied to solve the Reynolds averaged Navier-Stokes (RANS) equations. This method is based on a particular type of the residual weighting approach. In this approach, the computational zone is divided into finite control volumes as each node is covered by a control volume. Eventually, the differential equation is integrated on each finite volume [38–40].

Since in this study the particle volumetric loading ratio is below 10% (0%–4%), the DPM was applied for solving the diluted fluid-solid multiphase flow [41]. As such, the continuous phase, fluid, was simulated by utilizing the Eulerian approach whereas Lagrangian approach was used for modeling the particle phase. Standard wall functions were selected along with standard *k*-*ɛ* model described above.

The second-order upwind method [42–44] was chosen for the discretization of all terms, while the SIMPLEC algorithm (SIMPLE-Consistent) [15, 45, 46] was employed for pressure-velocity coupling. The impact angle function was specified utilizing a piecewise linear profile as per Table 2. The velocity exponent function and diameter function were fixed at 2.6 and 1.8 × 10^−09^, respectively, following [11]. The solution was converged when the residuals for all the equations dropped below 10^−6^ [38, 47].

## 5. Numerical Procedure Validation 

### 5.1. Validation with Numerical Study

**Figure 2:**
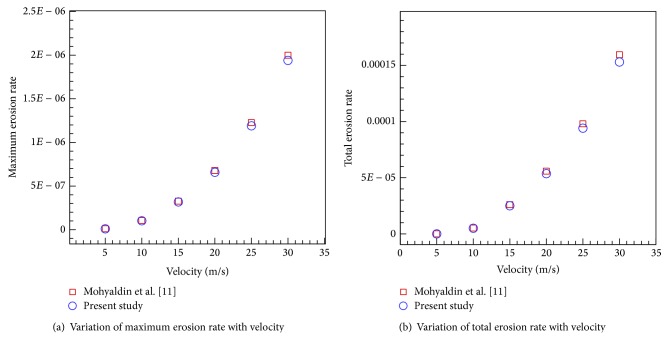
Comparison of total erosion rate and maximum erosion rate predicted here with those of [11].

In order to verify the present simulation, the results from this work were compared with those of [11] where sand erosion in a 2-D elbow was simulated. The geometry was a 50 mm diameter elbow with two 100 mm straight pipes protruded from both sides. The two-phase (air/sand) dilute slurry flow with sand as the dispersed phase was injected at 0.000886 kg/s to the continuous phase, here air, with an inlet velocity of 20 m/s. The variations of total erosion rate and maximum erosion rate with velocity were compared with the results reported by Mohyaldin et al. [11], as shown in Figures 2(a) and 2(b), to observe an excellent agreement between the results.

### 5.2. Validation with Experimental Study

**Figure 3:**
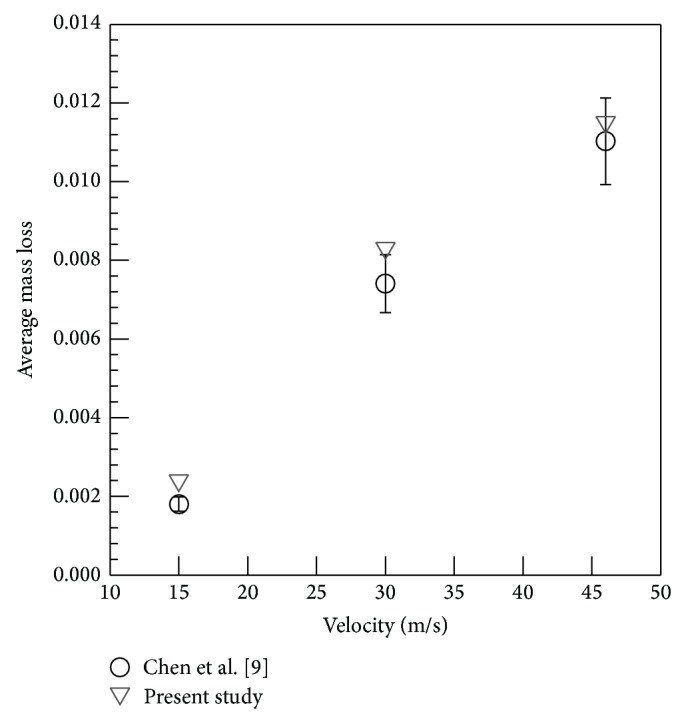
Comparison of average mass loss variations with previous work.

The numerical predictions based on our work were also compared with numerical and experimental results reported by Chen et al. [9] for erosion in elbows and plugged tees. Comparisons were performed for a 2.54 cm (diameter) elbow with a curvature ratio of 1.5 where sand particles of 150-micron diameter are injected at 2.08 × 10^−4^ kg/s over a range of air/sand velocities: 15.24, 30.48, and 45.72 m/s. The computed average mass loss for elbow was successfully compared with measurements reported in Chen et al. [9], as shown in Figure 3.

## 6. Grid Independence

The computational zone was discretized through structured, nonuniform hexahedral grid distributions. The refined grid was used at the vicinity of the walls where sharp gradients are expected. Several grid distributions were examined as Table 3 indicates. As seen, the effect of grid refinement beyond 61440 grids on the average erosion rate is insignificant implying grid independence of our results.

## 7. Results and Discussion

In this work, the turbulent fluid flow of water and copper micro- and nanoparticle suspensions through a 90° elbow has been investigated. The material of the 0.0032 m (1/8 inches) diameter elbow was aluminum (3003 Alloy). The length of the two attached pipe pieces at the beginning and the end of the elbow was 0.016 m (5/8 inches) long (5 times pipe diameter). The ratio of the bend radius to pipe inside diameter is equal to 1.5. Water was allowed to flow through the pipe at different velocities (5 m/s, 10 m/s, and 20 m/s). It was assumed that the solid particles are spherical and flow at the same velocity as that of water. Different particle diameters (10, 50, and 100 microns as well as 10, 50, and 100 nanometers) and particle volume fractions (2% and 4%) in the suspension were examined.

### 7.1. The Influence of Velocity on Erosion Rate

**Figure 4:**
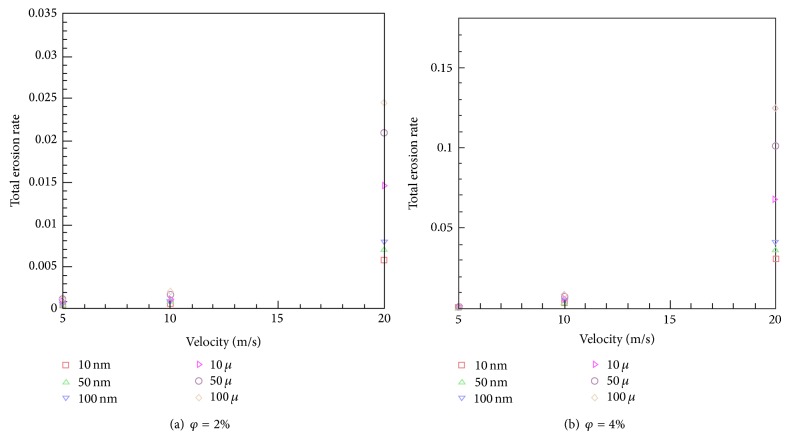
The variation of total erosion rate with velocity.

To investigate the impact of velocity on the maximum erosion rate and total erosion rate, several inlet velocities were simulated. The impact of inlet flow velocity on the total erosion rate is demonstrated in Figures 4(a) and 4(b) for different particle sizes. One notes that the total erosion rates are near zero for inlet velocity less than 5 m/s and particle volume fraction of 2%. For volume fraction of 4%, this quantity is still negligible for inlet velocity less than 5 m/s and particle diameters below 10 microns. This inlet velocity value of 5 m/s can be considered as a “threshold limit” for total erosion rate beyond which the total erosion rate rockets up with an increase in the inlet flow velocity for each particle diameter. These figures also indicate that, with the increase of particle volume fraction, the total erosion rate increases. The maximum of this erosion increase for *φ* = 4% is around 4.9 times at *V* = 20 m/s and *d*_*p*_ = 100 microns, compared to that of *φ* = 2%.

**Figure 5:**
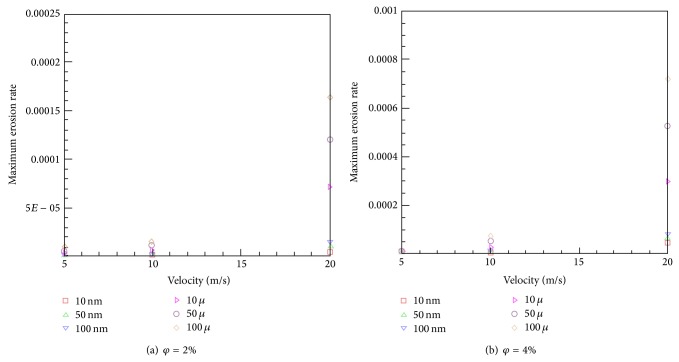
The variation of maximum erosion rate with velocity.

Similar trends are observed in Figures 5(a) and 5(b) for the maximum erosion rate at six various particle diameters. As seen, the maximum erosion rate is amplified with the particle diameter and velocity increment. This augmentation is negligible at velocities less than 5 m/s, but the difference between the values is more pronounced with an increase in the inlet velocity. Thus, when velocity is increased from 10 m/s to 20 m/s, the maximum erosion rate increases by about an order of magnitude, in fact, by around 7.5 times and 9 times at *φ* = 2% and *φ* = 4%, respectively.

### 7.2. The Effect of Particle Dimension on Erosion Rate

It is significant to study the effect of particle diameter on fluid-solid interaction as particles' size in different systems varies to a large extent from nanometer to centimeter. The particle diameter has direct influence on the drag force and, therefore, affects the flow behavior. The influence of particle diameter on maximum erosion rate, total erosion, pressure drop, and friction factor was studied by changing the particle diameter from 10 nm to 100 *μ*m.

**Figure 6:**
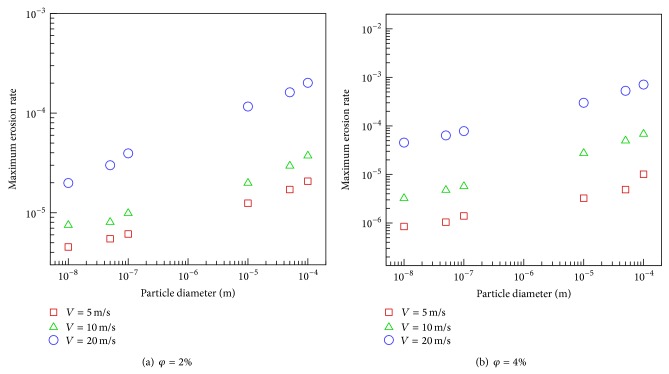
The variation of particle size with maximum erosion rate.

The influence of particle size on the maximum erosion rate was represented in Figures 6(a) and 6(b). As seen, the maximum erosion rate is closely related to the fluid velocity where a threshold velocity as well as a threshold particle size can be identified below which erosion is negligible. These figures also indicate that the rate of erosion augments linearly with particle diameter. One also notes that increasing the volume fraction of the particles, with other parameters fixed, will cause higher maximum erosion rate. The average of this increment is around 4.5 times.

**Figure 7:**
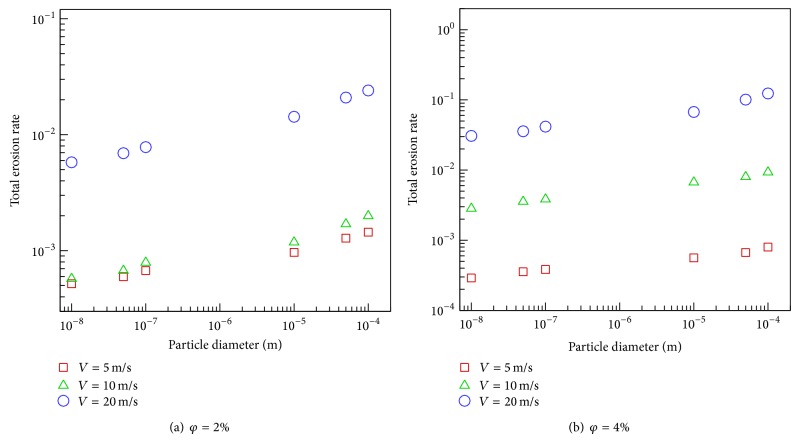
The variation of particle size with total erosion rate.

Similar trends are observed in Figures 7(a) and 7(b) for total erosion rate where higher erosion rate is observed when the particle diameter and inlet fluid velocity are increased. This is expected as the particle impact velocity grows with the increase of the inlet flow velocity and particle size (see (12)). However, our numerical results can be used to quantify this increment. Note that the increase in the total erosion rate is around 8.5 times for the increase of velocity from 10 m/s to 20 m/s at *φ* = 2% and 9.5 times at *φ* = 4%. The influence of volume fraction enhancement on total erosion rate is also around 8 times when the volume fraction is increased from 2% to 4%.

**Figure 8:**
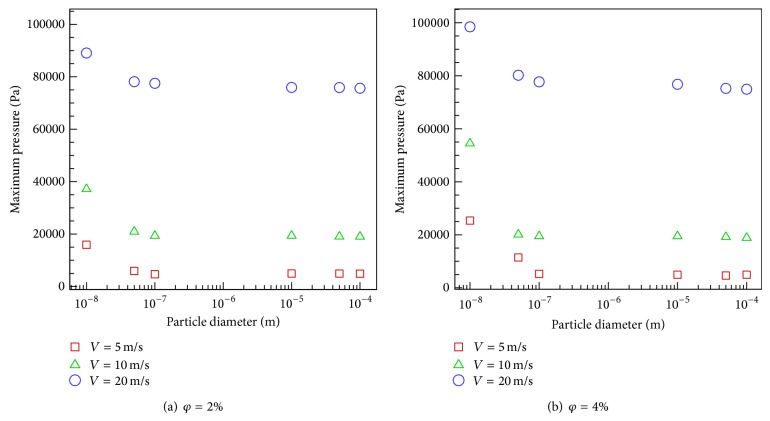
The variation of particle size with maximum pressure.

The declining impact of particle size on the maximum pressure was shown in Figures 8(a) and 8(b). This can be attributed to the reduction in drag forces as a result of an increase in the particle size. Consequently, with the same particle volume fraction, particle numbers are lowered compared to the case with smaller particles. The figures also indicate that there is a direct relationship between the velocity and increase of maximum pressure. It is also clear from the figures that an increase in particle volume fraction leads to higher maximum pressure. As a result, the maximum pressure value is observed when 10 nm particles at 4% volume fraction flow with water at 20 m/s.

**Figure 9:**
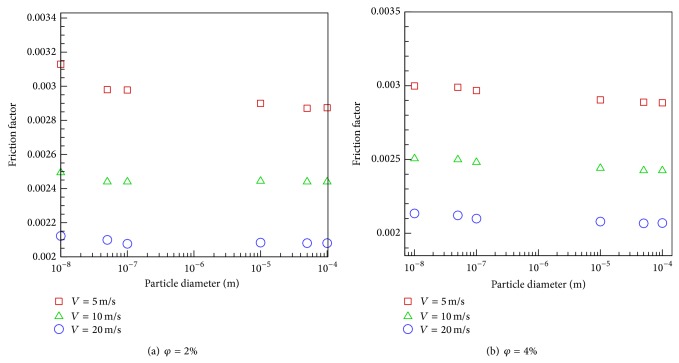
The variation of particle size with average friction factor.

Interestingly, according to Figures 9(a) and 9(b), the average friction factor—which has been calculated based on Fanning equation—is insensitive to either the particle size or volume fraction. However, one observes that the average friction factor increases with inlet velocity unlike a single-phase flow.

**Figure 10:**
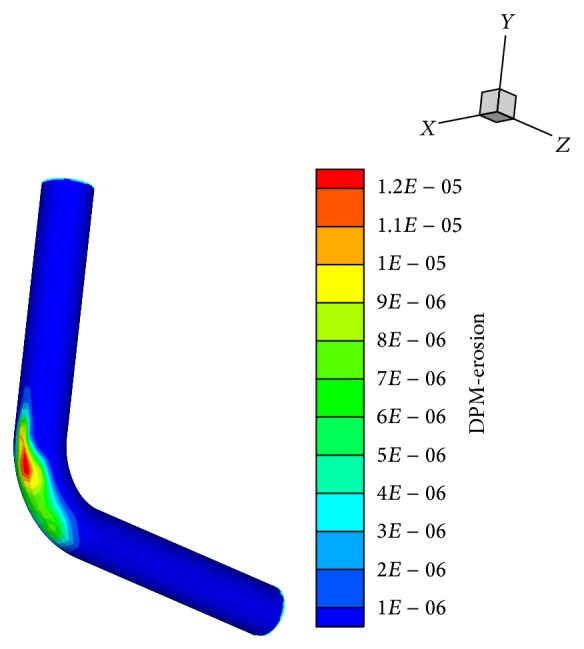
Erosion contour on the wall of the bend.

Figure 10 illustrates the erosion contour inside the elbow for *V* = 20 m/s, particle size = 100 microns, and the volume fractions of (Cu) 2%. As seen, the maximum erosion is observed near the midpoint, along the symmetry plane of the pipe bend, which is the location where velocity profiles begin an inverse behavior and the pressure is maximum.

**Figure 11:**
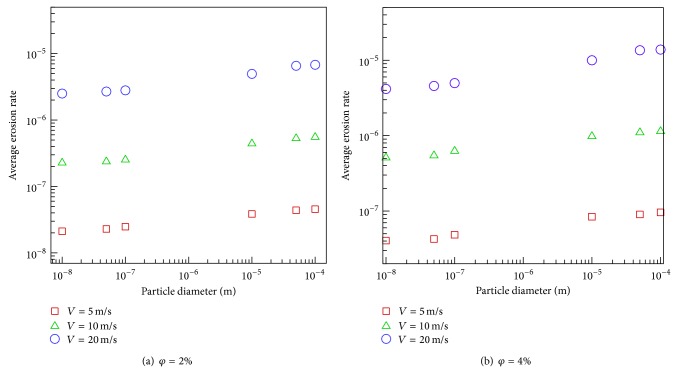
The variation of particle size with average erosion rate.

Finally, for engineering applications and presentation of the physical influence of the parameters, the following single nonlinear correlation is derived from Figures 11(a) and 11(b) to estimate the average erosion rate as a function of particles' concentration, diameter, and inlet velocity, valid for the range of parameters in this work; that is, 0.02 ≤ *φ* ≤ 0.04, 5 m/s ≤*V* ≤ 20 m/s, and 10 nm ≤*d*_*p*_ ≤ 100 microns. The average deviation of this correlation is 9.5%. Consider the following:

(13) Average  erosion  rate  (AER)  =3.6667×10−8(φ1.0024V3.4953dp0.1399).

## 8. Conclusion

A numerical study of erosion in turbulent water-based/copper (Cu) micro- and nanosized fluid flow through a 90° elbow has been conducted. Different solid volume fractions, particle sizes, and velocities were considered along with the maximum erosion rate, total erosion rate, average erosion rate, friction factor, and maximum pressure.

The conclusions are summarized as follows.

- There is a threshold velocity as well as a threshold particle size, beyond which erosion is significant.
- The maximum erosion rate, average erosion rate, and total erosion rate increase with particle diameter, volume fraction, and inlet fluid velocity.
- Increase of the particle diameter decreases the maximum pressure.
- An increase in particle volume fraction or velocity augments the maximum pressure.
- The average friction factor does not depend on particle size and/or volume fraction for a given flow rate.
- With the increase of the inlet velocity, the average friction factor enhances.

The usage of nanofluids in heat transfer has an obvious benefit from the thermal efficiency point of view. Nonetheless, care must be taken as depending on particle size, fluid velocity, particle shape, particle sedimentation, particle agglomeration, and surface erosion adverse effects can negate the benefits associated with heat transfer augmentation.

## Figures and Tables

**Table 1:** Coefficients for standard *k*-*ε* turbulent model.

*C*_μ_	σ_*k*_	σ_ε_	*C*_ε1_	*C*_ε2_
0.09	1	1.3	1.44	1.92

**Table 2:** Point values for impact angle function [11].

Point	Angle	Value
1	0	0
2	20	0.8
3	30	1
4	45	0.5
5	90	0.4

**Table 3:** Grid independence tests.

Number of grids (*V* = 20 m/s, φ = 2%)	**30720**	**61440**	**122880**
Average erosion rate for 100 *μ*m particles	6.9523 × 10^−6^	6.7833 × 10^−6^	6.6965 × 10^−6^
Number of grids (*V* = 20 m/s, φ = 2%)	**30720**	**61440**	**122880**
Average erosion rate for 10 nm particles	2.6789 × 10^−6^	2.5029 × 10^−6^	2.4351 × 10^−6^
Number of grids (*V* = 20 m/s, φ = 4%)	**30720**	**61440**	**122880**
Average erosion rate for 100 *μ*m particles	1.5270 × 10^−5^	1.3857 × 10^−5^	1.2994 × 10^−5^
Number of grids (*V* = 20 m/s, φ = 4%)	**30720**	**61440**	**122880**
Average erosion rate for 10 nm particles	4.3001 × 10^−6^	4.1646 × 10^−6^	4.0843 × 10^−6^

## Nomenclature

x, y: Cartesian coordinates (m)
d: Diameter (m)
$G_{k}$: Generation of turbulent kinetic energy (m^2^ s^−2^)
$\vec{g}$: Gravitational acceleration (m s^−2^)
$m_{p}$: Particle mass (kg)
${\vec{v}}_{p}$: Particle velocity (m s^−1^)
P: Pressure (N m^−2^)
Re: Reynolds number ($V\;D\upsilon^{-1}$)
$d_{p}$: Solid particle diameter (m)
t: Time (sec)
k: Turbulence kinetic energy (m^2^ s^−2^)
$\vec{V}$: Velocities vector (m s^−1^).

### Greek Symbols

ρ: Density (kg m^−3^)
ε: Dissipation rate of turbulent kinetic energy (m^2^ s^−3^)
μ: Dynamic viscosity (Pa s)
$\sigma_{k}$: Effective Prandtl number for k
$\sigma_{\varepsilon }$: Effective Prandtl number for ε
υ: Kinematics viscosity (m^2^ s^−1^)
$\upsilon_{t}$: Turbulence eddy viscosity (m^2^ s^−1^)
φ: Volume fraction of particles.

### Subscripts

D: Drag
p: Particle
t: Turbulent.

## References

[1] Keating A., Nesic S. Prediction of two-phase erosion-corrosion in bends.

[2] Nešić S. (2006). Using computational fluid dynamics in combating erosion-corrosion. *Chemical Engineering Science*.

[3] Southard J., Young R. A., Hollister C. D. (1971). Experimental erosion of calcareous ooze. *Journal of Geophysical Research*.

[4] Lonsdale P., Southard J. B. (1974). Experimental erosion of North Pacific red clay. *Marine Geology*.

[5] Gulden M. E. (1981). Correlation of experimental erosion data with elastic—plastic impact models. *Journal of the American Ceramic Society*.

[6] Saravanan R. A., Surappa M. K., Pramila Bai B. N. (1997). Erosion of A356 Al-SiCp composites due to multiple particle impact. *Wear*.

[7] Burstein G. T., Sasaki K. (2000). Effect of impact angle on the slurry erosion-corrosion of 304L stainless steel. *Wear*.

[8] Ahmed W. H., Bello M. M., El Nakla M., Al Sarkhi A. (2012). Flow and mass transfer downstream of an orifice under flow accelerated corrosion conditions. *Nuclear Engineering and Design*.

[9] Chen X., McLaury B. S., Shirazi S. A. (2004). Application and experimental validation of a computational fluid dynamics (CFD)-based erosion prediction model in elbows and plugged tees. *Computers and Fluids*.

[10] Gnanavelu A., Kapur N., Neville A., Flores J. F., Ghorbani N. (2011). A numerical investigation of a geometry independent integrated method to predict erosion rates in slurry erosion. *Wear*.

[11] Mohyaldin M. E., Elkhatib N., Ismail M. C. Evaluation of different modelling methods used for erosion prediction.

[12] Esfe M. H., Akbari M., Toghraie D., Karimipour A., Afrand M. (2014). Effect of nanofluid variable properties on mixed convection flow and heat transfer in an inclined two-sided lid-driven cavity with sinusoidal heating on sidewalls. *Heat Transfer Research*.

[13] Safaei M. R., Togun H., Vafai K., Kazi S. N., Badarudin A. (2014). Investigation of thermal conductivity and rheological properties of nanofluids containing graphene nanoplatelets. *Numerical Heat Transfer A*.

[14] Togun H., Safaei M. R., Sadri R., Kazi S., Badarudin A., Hooman K., Sadeghinezhad E. (2014). Numerical simulation of laminar to turbulent nanofluid flow and heat transfer over a backward-facing step. *Applied Mathematics and Computation*.

[15] Goodarzi M., Safaei M. R., Vafai K., Ahmadi G., Dahari M., Kazi S. N., Jomhari N. (2014). Investigation of nanofluid mixed convection in a shallow cavity using a two-phase mixture model. *International Journal of Thermal Sciences*.

[16] Routbort J., Singh D., Yu W., Chen G., Cookson D., Smith R., Sofu T. Effects of nanofluids on heavy vehicle cooling systems.

[17] Routbort J., Singh D., Timofeeva E., Yu W., Smith R. (2010). *Erosion of Radiator Materials by Nanofluids*.

[18] Routbort J., Singh D., Timofeeva E., Yu W., Smith R. (2011). *Erosion of Radiator Materialsby Nanofluids*.

[19] Karimipour A., Esfe M. H., Safaei M. R., Semiromi D. T., Jafari S., Kazi S. N. (2014). Mixed convection of copper-water nanofluid in a shallow inclined lid driven cavity using the lattice Boltzmann method. *Physica A: Statistical Mechanics and its Applications*.

[20] Esfe M. H., Esforjani S. S. M., Akbari M., Karimipour A. (2014). Mixed-convection flow in a lid-driven square cavity filled with a nanofluid with variable properties: effect of the nanoparticle diameter and of the position of a hot obstacle. *Heat Transfer Research*.

[21] Badr H., Habib M., Ben-Mansour R., Said S. Effect of flow velocity and particle size on erosion in a pipe with sudden contraction.

[22] Badr H. M., Habib M. A., Ben-Mansour R., Said S. A. M. (2005). Numerical investigation of erosion threshold velocity in a pipe with sudden contraction. *Computers and Fluids*.

[23] Mazumder Q. H. (2012). Effect of liquid and gas velocities on magnitude and location of maximum erosion in U-bend. *Open Journal of Fluid Dynamics*.

[24] Safaei M. R., Goshayeshi H. R., Razavi B. S., Goodarzi M. (2011). Numerical investigation of laminar and turbulent mixed convection in a shallow water-filled enclosure by various turbulence methods. *Scientific Research and Essays*.

[25] Safaei M. R., Maghmoumi Y., Karimipour A. Numerical investigation of turbulence mixed convection heat transfer of water and drilling mud inside a square enclosure by finite volume method.

[26] Sistani E. (2010). PIV measurements around a rotating single gear partially submerged in oil within modelled SAAB gearbox. *Diploma Work-Department of Applied Mechanics*.

[27] Habib M. A., Ben-Mansour R., Badr H. M., Kabir M. E. (2008). Erosion and penetration rates of a pipe protruded in a sudden contraction. *Computers & Fluids*.

[28] Abdolkarimi V., Mohammadikhah R. (2013). CFD modeling of particulates erosive effect on a commercial scale pipeline bend. *ISRN Chemical Engineering*.

[29] Zhu H., Zhao H., Pan Q., Li X. (2014). Coupling analysis of fluid-structure interaction and flow erosion of gas-solid flow in elbow pipe. *Advances in Mechanical Engineering*.

[30] Lipsett M. G., Bhushan V. (2012). Modeling erosion wear rates in slurry flotation cells. *Journal of Failure Analysis and Prevention*.

[31] Habib M. A., Badr H. M., Said S. A. M., Ben-Mansour R., Al-Anizi S. S. (2006). Solid-particle erosion in the tube end of the tube sheet of a shell-and-tube heat exchanger. *International Journal for Numerical Methods in Fluids*.

[32] Zhu H., Lin Y., Feng G., Deng K., Kong X., Wang Q., Zeng D. (2013). Numerical analysis of flow erosion on sand discharge pipe in nitrogen drilling. *Advances in Mechanical Engineering*.

[33] Campos-Amezcua A., Gallegos-Muñoz A., Alejandro Romero C., Mazur-Czerwiec Z., Campos-Amezcua R. (2007). Numerical investigation of the solid particle erosion rate in a steam turbine nozzle. *Applied Thermal Engineering*.

[34] Frawley P., Corish J., Niven A., Geron M. (2009). Combination of CFD and DOE to analyse solid particle erosion in elbows. *International Journal of Computational Fluid Dynamics*.

[35] Badr H. M., Habib M. A., Ben-Mansour R., Said S. A. M., Al-Anizi S. S. (2006). Erosion in the tube entrance region of an air-cooled heat exchanger. *International Journal of Impact Engineering*.

[36] Habib M. A., Badr H. M., Ben-Mansour R., Kabir M. E. (2007). Erosion rate correlations of a pipe protruded in an abrupt pipe contraction. *International Journal of Impact Engineering*.

[37] Sun K., Lu L., Jin H. (2013). Modeling and numerical analysis of the solid particle erosion in curved ducts. *Abstract and Applied Analysis*.

[38] Karimipour A., Afrand M., Akbari M., Safaei M. (2012). Simulation of fluid flow and heat transfer in the inclined enclosure. *International Journal of Mechanical and Aerospace Engineering*.

[39] Patankar S. V. (1980). *Numerical Heat Transfer and Fluid Flow*.

[40] Safaei M. R., Rahmanian B., Goodarzi M. (2011). Numerical study of laminar mixed convection heat transfer of power-law non-Newtonian fluids in square enclosures by finite volume method. *International Journal of Physical Sciences*.

[41] Xu P., Wu Z., Mujumdar A. S., Yu B. (2009). Innovative hydrocyclone inlet designs to reduce erosion-induced wear in mineral dewatering processes. *Drying Technology*.

[42] Goodarzi M., Safaei M. R., Oztop H. F., Karimipour A., Sadeghinezhad E., Dahari M., Kazi S. N., Jomhari N. (2014). Numerical study of entropy generation due to coupled laminar and turbulent mixed convection and thermal radiation in an enclosure filled with a semitransparent medium. *The Scientific World Journal*.

[43] Goshayeshi H., Safaei M., Maghmoumi Y. Numerical simulation of unsteady turbulent and laminar mixed convection in rectangular enclosure with hot upper moving wall by finite volume method.

[44] Safaiy M. R., Saleh S. R., Goudarzi M. (2011). Numerical studies of laminar natural convection in a square cavity with orthogonal grid mesh by finite volume method. *International Journal of Advanced Design and Manufacturing Technology*.

[45] Goodarzi M., Safaei M. R., Karimipour A., Hooman K., Dahari M., Kazi S. N., Sadeghinezhad E. (2014). Comparison of the finite volume and lattice Boltzmann methods for solving natural convection heat transfer problems inside cavities and enclosures. *Abstract and Applied Analysis*.

[46] Safaiy M. R., Goshayeshi H. R. (2011). Numerical simulation of laminar and turbulent mixed convection in rectangular enclosure with hot upper moving wall. *International Journal of Advanced Design and Manufacturing Technology*.

[47] Safaei M. R., Goodarzi M., Mohammadi M. (2011). Numerical modeling of turbulence mixed convection heat transfer in air filled enclosures by finite volume method. *The International Journal of Multiphysics*.

